# Report from the Western Canadian Gastrointestinal Consensus Cancer Conference—Management of Total Neoadjuvant Therapy in Rectal Cancer

**DOI:** 10.3390/curroncol29020078

**Published:** 2022-02-08

**Authors:** Howard Lim, Aswin George Abraham, Shahid Ahmed, Shahida Ahmed, Carl J. Brown, Bryan Brunet, Janine Davies, Corinne Doll, Dorie-Anna Dueck, Vallerie Gordon, Kimberly Hagel, Pamela Hebbard, Christina A. Kim, Duc Le, Richard Lee-Ying, John Paul McGhie, Karen Mulder, Jason Park, Daniel J. Renouf, Devin Schellenberg, Ralph P. W. Wong, Adnan Zaidi

**Affiliations:** 1 Department of Medical Oncology, British Columbia Cancer Agency, Vancouver, BC V5Z 4E6, Canada; Jan.davies@bccancer.bc.ca (J.D.); drenouf@bccancer.bc.ca (D.J.R.); 2 Cross Cancer Institute, Alberta Health Services, Edmonton, AB T6G 1Z2, Canada; aswin.abraham@albertahealthservices.ca (A.G.A.); Karen.Mulder@albertahealthservices.ca (K.M.); 3 Saskatoon Cancer Center, Saskatchewan Cancer Agency, Saskatoon, SK S7N 4H4, Canada; shahid.ahmed@saskcancer.ca (S.A.); Bryan.Brunet@saskcancer.ca (B.B.); Dorie-Anna.Dueck@saskcancer.ca (D.-A.D.); duc.le@saskcancer.ca (D.L.); adnan.zaidi@saskcancer.ca (A.Z.); 4 Department of Oncology, Cancer Care Manitoba, Winnipeg, MB R3E 0V9, Canada; sahmed1@cancercare.mb.ca (S.A.); vallerie.gordon@cancercare.mb.ca (V.G.); Pamela.Hebbard@cancercare.mb.ca (P.H.); Ckim3@cancercare.mb.ca (C.A.K.); ralph.wong@cancercare.mb.ca (R.P.W.W.); 5 Department of Surgery, University of British Columbia, Vancouver, BC V6T 1Z4, Canada; cbrown@providencehealth.bc.ca (C.J.B.); Jason.Park1@vch.ca (J.P.); 6 Tom Baker Cancer Center, Alberta Health Service, Calgary, AB T2N 4H2, Canada; Corinne.Doll@albertahealthservices.ca (C.D.); richard.lee-ying@ahs.ca (R.L.-Y.); 7 Department of Oncology, Allan Blair Cancer Centre, Regina, SK S4T 7T1, Canada; kimberly.hagel@saskcancer.ca; 8 Department of Medical Oncology, British Columbia Cancer Agency, Victoria, BC V8R 6V5, Canada; jmcghie@bccancer.bc.ca; 9 Department of Radiation Oncology, British Columbia Cancer Agency, Surrey, BC V3V 1Z2, Canada; dschellenberg@bccancer.bc.ca

**Keywords:** colorectal cancer, neo-adjuvant chemotherapy, surgery

## Abstract

An educational session related to the Western Canadian Gastrointestinal Cancer Consensus Conference (WCGCCC) was held virtually on 14 October 2020. The WCGCCC is an interactive multidisciplinary conference attended by health care professionals from across Western Canada (British Columbia, Alberta, Saskatchewan, and Manitoba), who are involved in the care of patients with gastrointestinal cancer. Surgical, medical, and radiation oncologists; pathologists, radiologists, and allied health care professionals participated in presentation and discussion sessions for the purpose of developing the recommendations presented here. This consensus statement addresses current issues in the management of total neoadjuvant therapy in rectal cancer.

## 1. Terms of Reference

### 1.1. Purpose

The aim of the Western Canadian Gastrointestinal Cancer Consensus Conference (WCGCC) is to develop the consensus opinion of oncologists and allied health professionals from across Western Canada, attempting to define the best care practices and to improve care and outcomes for patients with gastrointestinal cancers.

### 1.2. Participants

The WCGCC welcomes medical oncologists, radiation oncologists, surgical oncologists, pathologists, radiologists, gastroenterologists, and allied health professionals from western Canada who are involved in the care of patients with gastrointestinal malignancies.

### 1.3. Target Audience

The recommendations presented here are targeted to health care professionals involved in the care of patients with colorectal cancer (CRC).

### 1.4. Basis of Recommendations

The recommendations are based on the presentation and discussion of the best available evidence. Where applicable, references are cited.

**Which patients should be considered for TNT and what type of TNT—short versus long course of radiation, sequencing and type of chemotherapy, local excision for early rectal canc** **er.**

TNT is an evolving approach for rectal cancers and can be considered as one of the standards of care.

All patients should be discussed at multidisciplinary rounds which should include radiology, pathology, medical oncology, radiation oncology, and surgery. A staging MRI should be performed and reported in a synoptic standard. It can be used for patients with a good performance status with T4, node-positive disease, EMVI, involved mesorectal fascia and/or enlarged lateral lymph nodes, and patients with low rectal cancers at risk for APR. It can be used in select cases for patients with a less bulky disease.

Oxaliplatin-based chemotherapy is the favored systemic therapy approach in TNT and, in select cases, the addition of irinotecan can be considered. Both long-course and short-course radiation are reasonable options in the TNT approach, and may be tailored based on tumor and patient factors.

The optimal sequencing can be short-course or long-course chemoradiation prior to consolidative chemotherapy, as this leads to an improved pathologic complete response (CR).

Patients should undergo standard oncologic surgery for rectal cancer with the standard surveillance strategy. In the case of a clinical CR, a surveillance strategy can be considered in the context of a clinical trial.

Long-term data are still pending and a final approach is still to be determined.

In select patients with early-stage rectal cancer, a reasonable alternative could be local excision with neoadjuvant therapy. These patients would also need to undergo the intensive surveillance program with early salvage therapy for recurrent disease.

Patients should be considered for a clinical trial.

## 2. Summary of the Evidence

Treatment options for rectal cancer involve a multi-disciplinary review with pathology, radiology, surgery, radiation oncology, and medical oncology. Pre-operative treatment with either short-course radiation (5 × 5 Gy) or long-course radiation (50.4 Gy) over 5 weeks with concurrent capecitabine or 5-fluorouracil should be considered for Stage II or III tumors. After surgical resection, further adjuvant chemotherapy is considered based on clinical and pathological factors. Total neoadjuvant therapy comprising of radiation, chemoradiation, and pre-operative chemotherapy was investigated in several randomized phase III trials and could serve as an alternative in select patients.

The RAPIDO trial was a randomized open-label phase III trial [1] of 912 patients with biopsies proving locally advanced rectal adenocarcinoma (as defined as cT4a/b, extramural vascular invasion, clinical nodal stage N2, involving mesorectal fascia or enlarged lateral lymph nodes), who were randomized to short-course radiotherapy followed by six cycles of capecitabine and oxaliplatin (CAPOX) chemotherapy or nine cycles of infusional 5FU and oxaliplatin (FOLFOX) chemotherapy versus the standard of care. The standard of care was long-course chemoradiation of 50–50.4 Gy with capecitabine, followed by surgery. Adjuvant chemotherapy with 8 cycles of CAPOX or 12 cycles of FOLFOX were considered. At three years, 23.7% of patients in the experimental group versus 30.4% in the standard of care group had a cumulative probability of disease-related treatment failure, and a hazard ratio of 0.75, *p* = 0.19. Serious adverse events occurred in 38% of the experimental group versus 34% in the standard of care group. In the standard of care group, adverse events remained at 34% in both patients who received (64/187 patients) or did not receive adjuvant treatment (87/254 patients).

The OPRA trial randomized patients with MRI stage II and III rectal adenocarcinoma to 4 months of induction (before chemoradiation) or consolidation (after chemoradiation) fluropyrimidine and oxaliplatin-based treatment with fluropyrimidine and radiation [2] Patients were restaged 8–12 weeks after completion of therapy with a digital rectal exam (DRE), flexible sigmoidoscopy, and MRI. Patients with a complete or near-complete clinical response were offered a wait-and-watch approach; patients with an incomplete response had total mesorectal excision. Three hundred and twenty-four patients were enrolled and three hundred and seven patients were available for analysis. The compliance for chemotherapy was 82% and 81% for the induction and consolidation arms, respectively. The median radiation dose was 5400 cGY for both arms. The 3-year disease-free survival (78% vs. 77%, *p* = 0.90) and distant metastasis-free survival (81% vs. 83%, *p* = 0.86) were similar between induction and consolidation, respectively. Organ preservation rates were higher in the consolidation arm, 58% vs. 43%, *p* = 0.01. The watch-and-wait protocol comprised sigmoidoscopy DRE and CEA every 4 months for 2 years, then every 6 months for 3 years. A rectal MRI was performed every 6 months every 2 years and yearly for 3 years. Chest, abdomen, and pelvis CTs were performed yearly for 5 years and a colonoscopy was performed 1 year after diagnosis and per guidelines afterwards.

PRODIGE 23 was a randomized phase III trial involving 431 patients with cT3 or T4 rectal adenocarcinoma [3]. Patients received pre-op radiation 50Gy with capecitabine, followed by adjuvant chemotherapy for 6 months or six cycles of mFOLFIRINOX (oxaliplatin 85 mg/m^2^, leucovorin 400 mg/m^2^, irinotecan 180- mg/m^2^ D1, and 5FU 2400 mg/m^2^ over 46 h) every 14 days, followed by the same pre-operative chemoradiation therapy, followed by surgery, and then 3 months of adjuvant chemotherapy. Adjuvant chemotherapy was either mFOLFOX or capecitabine depending on the center’s choice. The ypT0N0 or complete pathologic response rate was 11.7% in the standard treatment group versus 27.5% in the experimental group treated with mFOLFIRINOX (*p* < 0.001). The three-year disease-free survival also increased, 68.5% (CI: 61.9–74.2) vs. 75.7% (CI: 69.4–80.8) (HR 0.69, 95% CI 0.49–0.97, *p* = 0.034), in favor of the FOLFIRINOX arm. The three-year metastasis-free survival improved, 71.7% vs. 78.8% (HR 0.64, CI 0.44–0.93, *p* < 0.02), and the three-year overall survival was 87.7 vs. 90.8% (HR 0.65, CI 0.40–1.05, *p* = 0.077) in favor of the mFOLFIRINOX arm.

The GRECCAR2 trial was a phase III trial that randomized patients with stage T2 or T3 lower rectal carcinoma with a good clinical response to neoadjuvant chemoradiotherapy (residual tumor < 2 cm), local excision, or total mesotectal excision [4] In the local excision group, a completion total mesorectal excision was performed if the tumor stage was ypT2-3. In total, 186 patients underwent chemoradiotherapy and 148 good clinical responders were randomized: 74 in the local excision group and 71 in the total mesorectal excision group. In the local excision group, 26 patients underwent a completion total mesorectal excision. The median follow-up was 60 months. The 5-year local recurrence rates were 7% in both groups, *p* = 0.60. Likewise, 18% of patients in the local excision group compared to 19% of patients in the total mesorectal excision group developed metastatic disease, *p* = 0.73. Disease-free survival at 5 years was 70% for local excision and 72% for total mesorectal excision (*p* = 0.68), and the 5-year overall survival was 84% for local excision and 82% for total mesorectal excision (*p* = 0.85).

## 3. Conclusions

TNT represents a new option for rectal cancer patients. This consensus statement addresses current issues in the management of total neoadjuvant therapy in rectal cancer.
